# Enablers and challenges of spatial omics, a melting pot of technologies

**DOI:** 10.15252/msb.202110571

**Published:** 2023-10-16

**Authors:** Theodore Alexandrov, Julio Saez‐Rodriguez, Sinem K Saka

**Affiliations:** ^1^ Structural and Computational Biology Unit European Molecular Biology Laboratory Heidelberg Germany; ^2^ Molecular Medicine Partnership Unit European Molecular Biology Laboratory Heidelberg Germany; ^3^ BioInnovation Institute Copenhagen Denmark; ^4^ Faculty of Medicine and Heidelberg University Hospital, Institute for Computational Biomedicine Heidelberg University Heidelberg Germany; ^5^ Genome Biology Unit European Molecular Biology Laboratory Heidelberg Germany

**Keywords:** genomics, metabolomics, proteomics, spatial omics, transcriptomics, Chromatin, Transcription & Genomics, Methods & Resources, Proteomics

## Abstract

Spatial omics has emerged as a rapidly growing and fruitful field with hundreds of publications presenting novel methods for obtaining spatially resolved information for any omics data type on spatial scales ranging from subcellular to organismal. From a technology development perspective, spatial omics is a highly interdisciplinary field that integrates imaging and omics, spatial and molecular analyses, sequencing and mass spectrometry, and image analysis and bioinformatics. The emergence of this field has not only opened a window into spatial biology, but also created multiple novel opportunities, questions, and challenges for method developers. Here, we provide the perspective of technology developers on what makes the spatial omics field unique. After providing a brief overview of the state of the art, we discuss technological enablers and challenges and present our vision about the future applications and impact of this melting pot.

## Graphical abstract

This Review discusses the spatial omics field from the point of view of technology developers. It provides an overview of the state of the art, discusses technological enablers and challenges and presents a vision about the future applications and impact of spatial omics technologies.

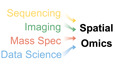

## Introduction

Biology is driven by interactions of molecules and cells with each other. Gaining insights into the intricate yet robust spatial regulation of biological processes, at the scales of organelles, cells, tissues, organs, and organisms, is key to understanding fundamental mechanisms of life. Furthermore, understanding the spatial context of the alterations of molecular and cellular programs during disease *in situ*, *in toto*, and *in vivo* is critical for diagnostics, developing novel and safe drugs, and discovering therapies for currently uncurable diseases.

The field of omics holds great potential to provide information‐rich readouts of molecular programs across all levels of the central dogma. It is currently undergoing an explosion with respect to spatial methods development, with the most prominent example being spatial transcriptomics, which was recognized as “the method of the year 2020” (Larsson *et al*, 2021; Marx, 2021; Zhuang, 2021). This rapid technological progress is notable across various omics modalities: genomics, epigenomics, proteomics, metabolomics, and fluxomics. The emerging field of spatial omics is now at a tipping point as the technology steps outside the technology developers' laboratories and spreads rapidly and widely, finding many applications in biology and molecular medicine.

In this Perspective, we discuss the origins and developments, the current drivers and challenges as well as the future potential of spatial omics from a viewpoint of experimental and computational technology developers. We do not aim to provide a comprehensive review of the state of the art of technologies and approaches in the field, for which we point the reader to recent excellent reviews (Rao *et al*, 2021; Moffitt *et al*, 2022; Moses & Pachter, 2022; Palla *et al*, 2022a; Vandereyken *et al*, 2023), but rather to highlight unique interdisciplinary aspects of the field and stimulate further cross‐fertilization.

## Where do spatial omics stand? An overview of the available technologies

**Figure 1:**
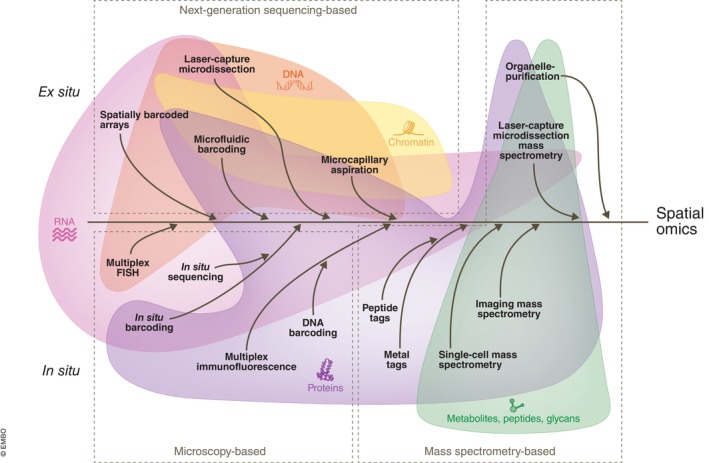
Evolutionary path of spatial omics technologies: unraveling the convergence Core capabilities stemming from fields of traditional omics, microscopy and mass spectrometry have led to emergence of diverse methods for spatial omics. Initially focused on only one molecular component, many of these approaches are increasingly getting combined to expanded repertoire of methods into multi‐omics domains.

Spatial omics methods are evolving at the intersection of two emerging directions: (i) *in situ* approaches that build on highly multiplex fluorescence microscopy or imaging mass spectrometry and (ii) *ex situ approaches* based on either next‐generation sequencing or mass spectrometry (Fig 1).

### *In situ* approaches

#### Transcriptomics, proteomics, genomics, epigenomics using highly multiplex fluorescence microscopy

Fluorescence microscopy has been for decades a go‐to approach in cell biology, for observing target biomolecules with high resolution in live and fixed cells. Fluorescence can be used for spatial mapping of gene expression (e.g., by RNA‐FISH) as well as localization of proteins (e.g., by immunofluorescence) and metabolites (e.g., using fluorescent metabolite sensors) in subcellular compartments, cells, tissues, and even whole organisms. The wish to increase the number of targeted molecules has been clashing with the limits of the spectral overlap. This limitation led to exploring various methods of *in situ* labeling to increase multiplexing, including: (i) hyperspectral imaging that utilizes a broader spectrum from fluorescence to Raman signatures (Wei *et al*, 2017; Du *et al*, 2020) particularly for spatial metabolomics or spectral unmixing in which spectral overlaps can be minimized or are resolved computationally (Gerner *et al*, 2012; Mehta *et al*, 2018; Seo *et al*, 2022; Lin *et al*, 2023), or (ii) cyclic labeling or cyclic imaging. For protein detection, traditional immunofluorescence methods were expanded by applying antibodies in cycles of staining and bleaching/elution (Gerdes *et al*, 2013; Lin *et al*, 2015, 2018; Gut *et al*, 2018). Furthermore, the use of nucleic acids offered multiplexing options through orthogonal DNA barcodes (Schueder *et al*, 2020), allowing simultaneous application of many antibodies and fast detection cycles with programmed hybridization/dehybridization of fluorescent oligos (Wang *et al*, 2012, 2017; Jungmann *et al*, 2014; Agasti *et al*, 2017; Schueder *et al*, 2017; Saka *et al*, 2019; Schürch *et al*, 2020).

These probe–erase–relabel approaches were better suited for linearly increasing the multiplexed detection of proteins, which show complex and overlapping spatial localizations and high dynamic range of expression. On the contrary, the more discrete spotty localization of RNAs allowed exponentially increasing the multiplicity of target detection through combinatorial barcoding. Combining RNA‐FISH with similar iterative detection cycles facilitated scaling multiplexing to 1000s of targets with methods such as SeqFISH and MERFISH (Lubeck *et al*, 2014; Chen *et al*, 2015; Moffitt *et al*, 2016; Eng *et al*, 2019; Xia *et al*, 2019b), and performing various approaches of cyclic decoding or *in situ* sequencing (Ke *et al*, 2013; Lee *et al*, 2014, 2022; Wang *et al*, 2018b; Gyllborg *et al*, 2020; Sountoulidis *et al*, 2020; Borm *et al*, 2023). Similar linear and exponential strategies were also applied in combination with DNA‐FISH to reveal new insights into spatial organization of chromatin and nuclear architecture (Beliveau *et al*, 2015; Wang *et al*, 2016; Nir *et al*, 2018; Mateo *et al*, 2019; Nguyen *et al*, 2020; Su *et al*, 2020; Takei *et al*, 2021b, 2021a, preprint: Takei *et al*, 2023). Moreover, incorporating combinatorial barcoding into a CUT&TAG‐like strategy (Bartlett *et al*, 2021), where epigenetic modifications of interest are labeled with antibodies and the associated genomics loci are detected by MERFISH after *in situ* transcription, allowed the spatial epigenomic profiling of hundreds of enhancers and promoters (Lu *et al*, 2022).

#### Proteomics, metabolomics, and lipidomics by using imaging mass spectrometry

In parallel, imaging mass spectrometry, where for a raster of pixels, a small amount of material from every pixel is desorbed, simultaneously ionized, and analyzed by mass spectrometry, has emerged as an indispensable and promising technology for *in situ* spatial mapping of proteins, peptides, metabolites, lipids, and glycans (Alexandrov, 2020). The most common approach in imaging mass spectrometry is matrix‐assisted laser desorption ionization (MALDI) imaging (Palmer *et al*, 2016). Numerous other approaches are available that do not use laser (Takáts *et al*, 2004). For spatial metabolomics applications, the current focus of imaging mass spectrometry is predominantly tissue cryosections, allowing for analyses of sections up to the size of a whole animal (Khatib‐Shahidi *et al*, 2006). The latest methods even demonstrate subcellular resolution (Niehaus *et al*, 2019; Pareek *et al*, 2020; Rovira‐Clavé *et al*, 2021). Untargeted detection of proteins in tissue sections with imaging mass spectrometry was demonstrated as early as 2001 (Stoeckli *et al*, 2001), with further improvements in resolution and speed over the years (Spraggins *et al*, 2016). Using imaging mass spectrometry in conjunction with metal‐tagged antibodies allowed reaching single‐cell level resolution for highly multiplexed targeted spatial proteomics (Angelo *et al*, 2014; Giesen *et al*, 2014). In parallel, using imaging mass spectrometry for metabolomics was boosted in the 2000s with the introduction of high‐resolution mass spectrometry analyzers. Imaging mass spectrometry is often used for detecting lipids due to the ease of sample preparation and detection of molecules of this class (Bowman *et al*, 2019). Recently, spatially resolved detection of N‐linked glycans attached to asparagine residues in proteins became possible, enabling spatial glycomics (McDowell *et al*, 2023). Imaging mass spectrometry was demonstrated to be an enabling method for spatial isotope tracing to spatially map metabolic activities as early as 2012 (Steinhauser *et al*, 2012; Louie *et al*, 2013). Later on, organismal‐scale fluxomics analyses using mass spectrometry revealed circulating lactate as an important energy source (Hui *et al*, 2017). And recently, imaging mass spectrometry was applied for high‐resolution spatial isotope tracing for cell‐type‐specific dynamics and metabolic activity in tissues (Wang *et al*, 2022a, 2022b).

### *Ex situ* approaches

#### Spatially resolved sequencing

*Ex situ* spatial sequencing methods are increasing in both numbers and diversity. In transcriptomics, the emergence of single‐cell RNA sequencing through droplet‐based separation of individual cells created opportunities for profiling cells by next‐generation sequencing (Klein *et al*, 2015; Macosko *et al*, 2015). Yet, these analyses were inherently unable to capture the spatial context of cells and to directly investigate cell–cell interactions (although indirect methods have been proposed, Armingol *et al*, 2021). This limitation led to a parallel exploration of ways to link spatial information with the sequencing data that are obtained *ex situ* when analyzing tissue sections. Several approaches were developed such as capturing RNA molecules on spatially barcoded arrays (Ståhl *et al*, 2016; Rodriques *et al*, 2019; Vickovic *et al*, 2019; Cho *et al*, 2021; Fu *et al*, 2022; Chen *et al*, 2022b), constructing positional DNA barcodes *in situ* by microfluidic delivery (Dbit‐Seq, Liu *et al*, 2020), photocrosslinking (Light‐Seq, Kishi *et al*, 2022) or photouncaging of spatial index oligos (Zip‐Seq, Hu *et al*, 2020), spatially confined collection of biomolecules or probes by microregion sequencing using light‐based cleavage for Digital Spatial Profiling (GeoMx, Merritt *et al*, 2020), and microdissection (LCM‐Seq (Nichterwitz *et al*, 2016), Image‐Seq (Haase *et al*, 2022)), mechanical isolation (Pick‐Seq, Maliga *et al*, 2021), or even extracting RNA from living cells by fluid force microscopy (Chen *et al*, 2022a).

Although most of these methods were initially applied to spatial transcriptomics, the majority could also be leveraged for spatial proteomics by incorporating readouts for DNA‐barcoded antibody libraries. Examples include the studies by Vickovic *et al* (2022), Liu *et al* (2023) and Ben‐Chetrit *et al* (2023). A subset of these ex situ strategies have been combined with *in situ* transposition to incorporate sequencing adapters into fixed genomic DNA, using Tn5 transposase similarly to ATAC‐Seq (Buenrostro *et al*, 2013) so that native spatial positions of accessible genomic DNA are preserved, enabling studies of spatial (epi)genomics (Chen *et al*, 2016b; Payne *et al*, 2020; Deng *et al*, 2022; Mangiameli *et al*, 2023).

Particularly for spatial transcriptomics, the parallel development *in situ* imaging or *ex situ* sequencing‐based methods, and novel combinations of workflows in these domains was critical for the quick expansion and success of the field (Larsson *et al*, 2021; Zhuang, 2021). It minimized risks, cross‐stimulated developments, and helped engage research groups with diverse backgrounds ranging from DNA biotechnology, microfluidics, sequencing, and microscopy to image analysis, and bioinformatics. Recently, these two branches began converging. On one hand, the resolution and sensitivity of the sequencing‐derived approaches are catching up with imaging (Vickovic *et al*, 2019; Cho *et al*, 2021; Stickels *et al*, 2021; Fu *et al*, 2022; Chen *et al*, 2022b). On the other hand, the multiplexing depth and throughput of imaging gets closer to omics level, including the latest FISH‐based techniques that can image the expression of 1000s of genes (Eng *et al*, 2019; Su *et al*, 2020; Takei *et al*, 2021b, 2021a). We foresee the FISH‐like targeted probe design strategies getting increasingly used to improve the sensitivity of sequencing‐based approaches, as an alternative to the more standard polyA‐capture, especially for more challenging samples such as FFPE preparations (for example, 10X Genomics Visium FFPE kit or Xenium system, or Nanostring GeoMx or CosMx systems).

#### *Ex situ* mass spectrometry

Although, as previously discussed, mass spectrometry is currently more popular for *in situ* analyses by means of imaging mass spectrometry, there are several notable *ex situ* approaches. Among them, laser‐capture microdissection is commonly used for extracting individual cells or small regions of interest from a tissue section for subsequent mass spectrometry analysis, as demonstrated for spatial proteomics (Zhu *et al*, 2018a,b; Mund *et al*, 2022) and spatial metabolomics already as early as in 2005 (Schad *et al*, 2005). Another group of *ex situ* methods often referred as spatial metabolomics is the proteome profiling of purified organelles or other cell compartments followed by mass spectrometry (Dunkley *et al*, 2004). Such methods enabled creating a subcellular map of the human proteome (Mulvey *et al*, 2017; Thul *et al*, 2017) and led to a proximity‐based mapping of the proteome of a human cell (Go *et al*, 2021). Lately, metabolomics was also applied for purified organelles (Chen *et al*, 2016a; Zhu *et al*, 2021). On another, opposite end of the spatial scales, spatial sampling followed by mass spectrometry was used for creating 3D molecular cartography maps at the organismal and supra‐organismal scales (Bouslimani *et al*, 2015; Petras *et al*, 2017).

### Spatial omics is empowered by bespoke computational methods

**Figure 2:**
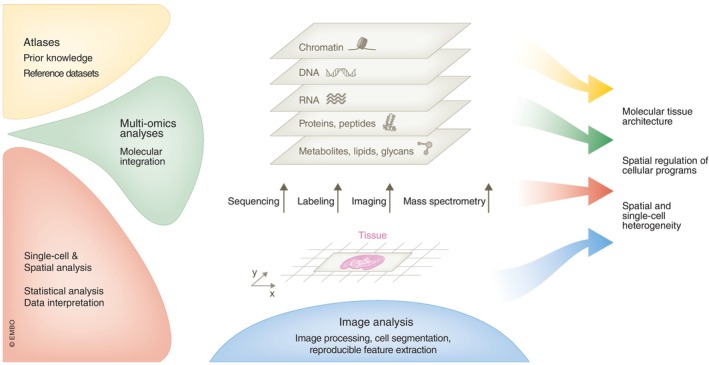
Unveiling new insights with spatial omics An overview of the data sources and knowledge inputs, computational approaches employed for data analysis, and pivotal questions central to spatial omics. The illustration outlines the individual omics layers, each providing a complementary view of the cellular programs.

These technological developments have been accompanied by a rapid evolution of computational methods (Fig 2). On one hand, image processing and high‐dimensional analysis approaches have brought handling of highly multiplex fluorescence microscopy data closer to omics data. Image data typically offer single‐cell or subcellular resolution. Cell segmentation was substantially improved with the incorporation of powerful deep learning approaches focused on highly multiplexed imaging (Greenwald *et al*, 2021; Yapp *et al*, 2022) to further incorporate information about the localization of cells and their neighbors in the tissue. Alternatively, segmentation‐free methods provide a different approach for dealing with images where cells are hard to segment. These approaches can assign molecules to cells based on the likelihood of particular transcriptional compositions and cell morphology by incorporating prior knowledge of cell types obtained from scRNA‐seq (Park *et al*, 2021; Petukhov *et al*, 2021).

Different methods have been developed that can identify spatial patterns in the expression of genes or correlations across genes, based on the resulting processed data (Edsgärd *et al*, 2018; Svensson *et al*, 2018; Ghazanfar *et al*, 2020; Sun *et al*, 2020). Numerous approaches for spatial data analyses have been developed, focused on different aspects: revealing interactions between the markers across different spatial contexts, investigating the distribution of identified cell types in the neighborhood of each cell (Schapiro *et al*, 2017; Goltsev *et al*, 2018; Keren *et al*, 2018), or decomposing the effect among markers at the cellular intrinsic, extrinsic, and intercellular levels (Arnol *et al*, 2019; Tanevski *et al*, 2022). Another emerging direction is the computational integration with single‐cell omics data. In transcriptomics, this helped tackle some limitations of spatial transcriptomics: first, by deconvolving cosampled cell types in spatially resolved sequencing with spot sizes encompassing 5–100 cells (Andersson *et al*, 2020; preprint: Kleshchevnikov *et al*, 2020; Cable *et al*, 2021; Elosua‐Bayes *et al*, 2021; Li *et al*, 2022) and second, by imputing unmeasured transcripts in targeted multiplex measurements (Biancalani *et al*, 2021; preprint: Rahimi *et al*, 2023). In parallel, computational approaches can estimate the location of single cells dissociated from the tissue (Bageritz *et al*, 2019; Nitzan *et al*, 2019; preprint: Biancalani *et al*, 2020) or map them via fiducial genes (Achim *et al*, 2015; Satija *et al*, 2015; Tanevski *et al*, 2020). Furthermore, methods have been developed to identify spatial domains with common molecular profiles and neighborhood structures (Hu *et al*, 2021; Zhao *et al*, 2021), and to study cell–cell interactions (Cang & Nie, 2020; Yuan & Bar‐Joseph, 2020; Garcia‐Alonso *et al*, 2022; Tanevski *et al*, 2022; Cang *et al*, 2023; Fischer *et al*, 2023).

Several software frameworks are available for analyzing spatially resolved data, such as Squidpy (Palla *et al*, 2022b), SpatialExperiment (Righelli *et al*, 2022), Giotto (Dries *et al*, 2021), and Seurat (preprint: Hao *et al*, 2022), which are primarily focused on spatial transcriptomics data, and MCMICRO (Schapiro *et al*, 2022a) for multiplexed protein data. These frameworks, which are continuously evolving, provide integrated suits that facilitate the application of state of the art methods (Heumos *et al*, 2023) for users without advanced computational skills.

## Enablers and challenges

Looking back at the recent history of spatial omics from our point of view as technology developers, we asked what were the key enablers that jump‐started technological developments in spatial omics at the fast pace, that was key for establishing this field and for contributing to its success.

### Pushing the technical limitations

When spatial omics started emerging, cyclic imaging paved the way to deepen the information content of microscopy experiments. The exploration and development of protocols that go beyond conventional fluorescent staining and imaging, and allow reprobing the same sample were crucial for imaging‐based multiplexing both for protein and RNA detection. For proteins, the use of direct primary antibody conjugations with fluorophores and alternative tags such as metals or DNA facilitated the creation of bigger panels. This also created a demand for sourcing of primary antibodies in formats that are more amenable to direct conjugation and creating kits for increasing the accessibility of the different protocols, as well as a higher scrutiny for antibody qualifications (Hickey *et al*, 2021).

For RNA and DNA detection, easy and cheaper access to synthetic oligos and the development of high‐fidelity FISH protocols utilizing probe tiling as in single molecule FISH (Femino *et al*, 1998; Orjalo *et al*, 2011; Beliveau *et al*, 2012) was important to attain a good signal‐to‐noise ratio while offering flexibility with probe design. Other *in situ* amplification approaches that have been transforming *in situ* RNA, DNA, and protein labeling include reactive probe deposition (Kerstens *et al*, 1995), DNA branching (Player *et al*, 2001; Wang *et al*, 2012; Kishi *et al*, 2019; Saka *et al*, 2019; Xia *et al*, 2019a), *in situ* polymerization (by rolling circle amplification (Lizardi *et al*, 1998; Lee *et al*, 2014; Nagendran & Riordan, 2018; Wang *et al*, 2018b; Liu *et al*, 2021), hybridization chain reaction (Dirks & Pierce, 2004; Choi *et al*, 2010, 2014, 2018; Shah *et al*, 2016; preprint: Wang *et al*, 2020)), or chemical ligation (Rouhanifard *et al*, 2018; Dardani *et al*, 2022).

Technical limitations in resolution, sensitivity, and throughput still constitute important barriers. In addition to signal amplification, the integration of super‐resolution (Jacquemet *et al*, 2020) and expansion microscopy approaches (Wen *et al*, 2023) with spatial omics methods start to provide valuable improvements in resolution and sensitivity for imaging‐based spatial omics (for example (Shah *et al*, 2017; Nir *et al*, 2018; Wang *et al*, 2018a; Eng *et al*, 2019; Saka *et al*, 2019; Nguyen *et al*, 2020; Su *et al*, 2020; Alon *et al*, 2021)). For methods that rely on DNA barcodes, advances in DNA barcoding protocols (both for reagents such as antibodies and cellular molecules) and barcoded array generation methods that can support even higher densities and smaller spots at lower costs will be important to improve the resolution and sensitivity (Cho *et al*, 2021; Chen *et al*, 2022b).

By now, DNA has become a go‐to barcoding molecule that helped bridge imaging and sequencing‐based approaches for spatial genomics, epigenomics, transcriptomics, and proteomics (Schueder *et al*, 2020). This success has been largely enabled by cost‐effective and reliable oligo synthesis (Hughes & Ellington, 2017) and the accumulated knowledge in DNA nanotechnology and computational tools and databases that improved our ability to *in silico* design nucleic acid probes with predictable kinetics (Zadeh *et al*, 2011; Beliveau *et al*, 2018; Mengwei *et al*, 2020; Passaro *et al*, 2020). It is foreseeable that more cost‐effective options for sequencing and DNA synthesis will further drive the progress and wider adoption of spatial omics in the future.

A large subset of spatial omics methods are not directly applicable for imaging larger volumes. Hence, performing spatial omics in 3D often requires serial sectioning with consecutive sections analyzed separately (Palmer & Alexandrov, 2015). Considering that the thickness of such sections can exceed the cell size, especially when performed on frozen tissue, this leads to discrepancies between sections that cannot be compensated. Methods that enable 3D spatial omics in thick samples (organoids, tissues, whole organs, and organisms), including tissue clearing and embedding approaches, reagent delivery and fluidics, new fast optical systems and image processing methods, hold great potential to be integrated with spatial omics. Some examples of interesting approaches in this direction include: Chung & Deisseroth (2013), Murray *et al* (2015), Pan *et al* (2016), Ku *et al* (2016), Wang *et al* (2018b), Park *et al* (2018), preprint: Choi *et al* (2019), Perens *et al* (2020) and Bhatia *et al* (2022).

The introduction of high‐resolution mass spectrometry (HRMS) was a key enabler for spatial metabolomics, lipidomics, and glycomics, which predominantly use imaging mass spectrometry (Palmer *et al*, 2016). The high spectral resolution enabled metabolite assignment with a substantially higher confidence even without the need for MS/MS fragmentation that is often impractical in spatial applications. Over the past years, all major imaging mass spectrometry vendors have introduced HRMS‐based instrumentation for spatial metabolomics analyses. We expect the use of imaging HRMS to continue supporting a rapid evolution of spatial metabolomics, lipidomics, and fluxomics. Nevertheless, the field still faces challenges including sensitivity, unwanted metabolite degradation and fragmentation, metabolite identification, and data interpretation that will likely drive future experimental and computational developments.

### Reproducibility and commercialization

Within the rapidly growing field of spatial omics with multiple technologies still being in the early phases of development, a key practical challenge is to increase the reproducibility and fidelity of the results. Minor differences in sample handling, probe preparations, staining protocols, and instrumentation can introduce unwanted variations in the results, particularly when it comes to coverage, background, and sensitivity. Standardizing protocols and performing comparative analyses is complicated, due to, for example, the wide range of possible parameters for protocol optimization (including sample fixation/preservation, permeabilization, oligo probe sequences, choice of MALDI mass spectrometry matrices, and ion polarity mode) and the unique alterations that may be necessary to get the best results for specific tissues or custom instrumental setups. The importance of such optimizations is revealed by the typically iterative method development process, with newer and significantly improved versions being developed as a result of seemingly small adjustments to the protocols (e.g., slide‐seq v1 vs. v2, SeqFISH vs. SeqFISH+, and MALDI vs. MALDI‐2). Compared with more mature omics approaches, spatial omics can be still considered at its infancy, meaning that most methods and protocols are at a relatively nascent state and are expected to be substantially improved as they become more widely used. Increasing access to automation, streamlining of advanced multistep technological workflows and a growing popularity of microfluidics, barcoding technologies, and imaging mass spectrometry have been improving the scalability and reproducibility of the methods and is expected to drive the field forward. Reliable and compact automation systems that can help screening of sample preparation conditions with custom protocols, while supporting different sample formats and delivering high reproducibility constitutes a high unmet need.

Commercialization of methods developed in academic laboratories has been a major driver of high‐pace method popularization and helped lower the entry barrier for new users who would like to use these methods for their research questions. In the last decade, we have witnessed a rapid adoption of methods where commercial end‐to‐end automation (e.g., Akoya CODEX platform, Nanostring GeoMx and COSMx systems, 10X Genomics Visium and Xenium, Resolve Biosciences Molecular Cartography, Fluidigm Hyperion Imaging System, IONpath MIBIScope, VIZGEN MERScope, Militenyi MACSima, Lunaphore COMET), optimized protocols and reagent kits (e.g., RNAscope), integrated microscopy and mass spectrometry instrumentation (e.g., Shimadzu iMScope) and data processing tools were made available. Importantly, this could not be have been achieved without academic feedback fueling constant improvement of the products and methods. Many new startups are currently entering the market with reagent kits, automated hardware solutions, software tools, AI/ML platforms, and service models that will create numerous future possibilities in establishing novel assays, data acquisition, and analysis. In a market that is getting increasingly competitive but still at an early stage in terms of scientific capabilities, it will be important to not lose this diversity of technologies (which, most often than not, offer complementary information) due to IP conflicts and lawsuits will be important to keep the field moving forward with the same speed and creative freedom and allow careful vetting and maturation of technologies and services by the scientific community.

From the academic standpoint, there would be some other critical concerns related to relying only on industrial development. From one hand, using end‐to‐end approaches and commercial reagents does in principle improve reproducibility and facilitates access to novel technologies. However, the need for using proprietary reagents, algorithms, workflows, formats, and closed‐box systems might in turn create barriers. Foremost, proprietary systems and reagents create a paywall that may cut off the wider scientific community from reproducing the data or utilizing it. Second, this barrier impedes noncommercial development, improvement and combination of the spatial omics methods. For example, lacking the knowledge of original barcodes or probe sequences in commercial kits may prevent scientists from creating new workflows that incorporate complementary methods for multi‐omics analysis. Similarly, the use of proprietary data formats prevents interoperability and locks users in the limited software ecosystem offered by a vendor, thus inhibiting uptake in the long term. Moreover, the overlapping development time line of similar methods in both academic and commercial setups may create a version confusion in which emerging data may be attributed to previously published versions of the methods and protocols. These may be different from the commercial workflow and expectedly modified during conversion of early academic intellectual property to marketable products, making it hard to trace data‐protocol links. This may constrain the systematic accumulation of the new knowledge and create reproducibility issues. Finally, the risk of monopoly creation and price inflation may contribute to increasing the already high prices of spatial omics methods and thus hamper their accessibility. In summary, although commercialization can drive broader adoption, increased access, utility, and reproducibility of these methods, it is important for the field to create and value a rich ecosystem of technologies and vendors, and to support the open and free exchange of protocols and software whenever possible.

Going forward, and to ensure a sustainable growth of the field, it will be critical to empower open science while fostering academic‐industry collaborations. This can help address the major practical challenges, including the cost and throughput of the approaches significantly limiting the scalability, and thus ensure wide‐spread access and translation of the spatial omics approaches.

### Data handling and computation

The development and application of high‐accuracy image segmentation methods as well as the adaptation of analysis tools originally utilized in single‐cell omics for spatial omics data have been instrumental during the early stages of the field. However, spatial omics data pose distinct computational challenges (Hériché *et al*, 2019; Alexandrov, 2020; Lähnemann *et al*, 2020) due to the added dimensions and increased data size, and the special nature of the data, which is often different to the bulk omics with respect to coverage, sensitivity, level of noise, and overall amount of represented information. Single‐cell‐focused tools also offer limited utility for leveraging new opportunities provided by the spatial content of the data.

The large size of spatial datasets imposes heavy requirements on data handling, infrastructure, and computational performance. Moreover, the unique aspects of spatial data including the spatial information, need for custom visualization, and often custom data formats require novel data frameworks and repositories focused on spatial omics data. Previously generated datasets and atlases such as Human Protein Atlas (Uhlén *et al*, 2015; Thul *et al*, 2017) or Allen Brain Atlases (Ortiz *et al*, 2020; Viana *et al*, 2023) have become very useful as references and frameworks for the spatial omics data to build on. Creating new repositories that can support deposition of both the raw and processed data will not only provide reference platforms but also enable benchmarking and data reprocessing and analysis with future computational tools. Ideally such spatial omics repositories shall embed advanced annotation, and analysis tools to collectively provide broader insights, as demonstrated by the METASPACE platform for spatial metabolomics (preprint: Alexandrov *et al*, 2019). For this a future‐proof implementation of unified nonproprietary data formats, annotations and metadata structures will be crucial. Importantly, increasing data size and complexity will likely require next‐generation data formats (Moore *et al*, 2023) and approaches for data management and analysis (such as preprint: Marconato *et al*, 2023; Walter *et al*, 2023).

Another challenge is the integration of multiple datasets from the same spatial omics technologies. The integration of sequencing‐based data into meta‐studies is a common practice. The integration of single‐cell transcriptomics data has been successfully demonstrated (Luecken *et al*, 2022; Argelaguet *et al*, 2021), as has been the combination of single‐cell atlases and spatially resolved datasets (Lohoff *et al*, 2022). However, the integration has been challenging for spatial omics datasets. The integration of image‐based data requires appropriate methods of registration and normalization, and the continuous nature of imaging data has to be taken into account. Another critical component is to have anchors that can link datasets, such as mapping one image to the other to be able to perform meta‐analyses. However, there are not yet established anchor points that can be used for normalizing, analyzing and comparing samples, limiting the utility of the data that are available in databases. The challenge is exacerbated if different data modalities are to be integrated—even if one measures the protein and transcript levels for the same genes, given the limited protein‐transcript correlation. We anticipate that addressing this challenge will be an active area of method development in the next few years. In this context, it will be essential to have adequate data and metadata standards (Schapiro *et al*, 2022b) as well as benchmarks (Lähnemann *et al*, 2020) to rigorously assess strengths and weaknesses of each method. As a means to leverage such benchmarks to accelerate the development of methods in an unbiased way, crowdsourced competitions (Tanevski *et al*, 2020) should be considered, complemented by a broader acceptance in funding benchmarking efforts.

Spatial omics research taps into a broad range of disciplines, which requires a higher level of interdisciplinarity and broader expertise. This creates a need for coordinated large‐scale efforts not only to generate and interpret the data but also to improve the technologies and make both the methods and the data more accessible and reproducible. In this regard, the attention of public and private funding bodies such as European Commission, National Institutes of Health, Chan Zuckerberg Initiative or Knut and Alice Wallenberg Foundation to create large consortia for developing and applying spatial omics approaches is critical. These consortia, including the Human Cell Atlas (HCA; Aviv *et al*, 2017), Human Biomolecular Atlas Program (HuBMAP; Aviv *et al*, 2017; HuBMAP Consortium, 2019), HTAN (Rozenblatt‐Rosen *et al*, 2020), KPMP (de Boer *et al*, 2021), LungMAP (Ardini‐Poleske *et al*, 2017), 4D nucleome (Dekker *et al*, 2017), and LifeTime (Rajewsky *et al*, 2020), have been instrumental in creating frameworks, standards, data repositories, analysis, and visualization tools as well as building a community of developers and users of spatial omics methods. Going forward, expanding such coordinated efforts and spreading them globally will tremendously improve the utility and accessibility of these methods. Such efforts will be critical to create the reference datasets and the infrastructure necessary to push spatial omics to the next level.

## The future evolution and impact of spatial omics

The spatial omics field is currently a melting pot in which contributions from various disciplines are explored, evaluated, integrated, and implemented, to create methods that offer higher resolution and sensitivity, and multiple omics layers and sources of molecular knowledge, while being cost‐efficient and robust. The future and promise of spatial omics are indisputably bright and bold. Here, we look beyond this field and hypothesize how spatial omics will enable other areas, from a high‐level technological perspective.

### Further technology development

Spatial omics is currently pushing technology development in other fields and, assuming its increased adoption and popularity, it is expected to have an even bigger and broader impact in related technological fields. It has already created an increased demand in barcoding approaches, microfluidics, and sequencing. Sequencing‐based approaches are often prohibitively expensive when applied to every pixel or spatial location, which will likely create further pressure to develop more cost‐effective sequencing methods. Furthermore, in single‐cell omics experiments, the choice of the specimen for single‐cell interrogation is often limited by the sampling method or specimen's anatomy. This bias does not necessarily provide a representative sample capturing the required heterogeneity, cell types, cell states, and phenotypes of interest. Here, spatial omics, once made accessible, can be a useful as a common preceding approach (much like flow cytometry enrichment of cell of interest before scRNA‐Seq) to provide guidance for more representative and informative selection of the samples (i.e., regions and cells of interest) for omics‐level analysis with NGS and mass spectrometry and in particular can enable interrogation of rare cell types and states. Similar to biomarker‐based presorting of the cells of interest for deeper sequencing, spatial omics could also be applied selectively only to regions/cells of interest to reduce the experimental costs and to enable classifying the cells at much higher depth, as shown by recent for spatially directed omics applications (Nichterwitz *et al*, 2016; Buczak *et al*, 2020; Hu *et al*, 2020; Merritt *et al*, 2020; preprint: Maliga *et al*, 2021; Kishi *et al*, 2022; Mund *et al*, 2022). Here, the development of novel barcoding technologies, integrated physical, or contextual spatial dissection capabilities, as well as microscopy‐driven mass spectrometry approaches can make performing such selection attainable at the single‐cell level and beyond.

#### Mass spectrometry

In mass spectrometry, the rise of spatial omics greatly stimulated the development of desorption approaches (MALDI, SIMS and DESI) that previously had a much narrower scope of applications. We will likely see more imaging mass spectrometry developments specifically focused on spatial omics applications to address the demand for higher mass resolution, sensitivity, and speed. Among key developments in this field are the transmission‐based MALDI desorption allowing for subcellular resolution (Niehaus *et al*, 2019), the increased use of fast analyzers such as QTOF, introduced ion mobility separation (IMS) for higher specificity and enhanced molecular identification, and modifications of ultrahigh‐resolution SIMS imaging mass spectrometry aiming to reduce unwanted fragmentation and allowing for detection of biomolecules (Pareek *et al*, 2020; Ganesh *et al*, 2021). Finally, the increase of sensitivity stimulated by the emerging single‐cell mass spectrometry capabilities by improving sample preparation, chromatography, mass spectrometry, and computational approaches will likely have a positive impact on spatial omics for *ex situ* but also for *in situ* approaches.

#### Sequencing‐based

Future adaptations of 3D chromatin conformation capture methods (3C, 4C, 5C, Hi‐C, Micro‐C, SPRITE and many more; Grob & Cavalli, 2018) that would go beyond the short and long‐range interactions and reveal the absolute or relative 3D spatial location of genetic sequences inside the nucleus down to single‐cell level would complement the multiplexed and combinatorial imaging‐based spatial genomics approaches (Payne *et al*, 2020; preprint: Takei *et al*, 2023) and greatly enhance our understanding of nuclear organization and gene regulation. Although still at its infancy, DNA microscopy and proximity detection approaches are also poised to create new capabilities in converting nanoscale spatial information into sequencable readouts (Schaus *et al*, 2017; Hoffecker *et al*, 2019; Weinstein *et al*, 2019; preprint: Gopalkrishnan *et al*, 2020).

#### Spatial multi‐omics

Beyond combining spatial location of cells and their neighborhoods with the cell types and states as typically defined by transcriptomics approaches, spatial omics is poised to integrate many more layers of information. As opposed to single‐cell approaches that operate on dissociated cells that are typically lysed/desorbed/consumed in the process, spatial omics approaches can more easily support spatial multi‐omics by applying different modalities on the same cells (Vickovic *et al*, 2022; Liu *et al*, 2023; preprint: Takei *et al*, 2023; Vandereyken *et al*, 2023; Zhang *et al*, 2023). For many of the existing approaches, it is a matter of optimization of the sample preparation conditions to copreserve the molecular composition and detection access for multiple components (RNA, protein, and DNA) simultaneously and more combinations will no doubt be increasingly utilized for high‐throughput experiments (Baysoy *et al*, 2023).

Mass spectrometry also provides multiple capabilities. In general, small molecules, lipids, glycans, and small peptides can be detected by employing broad mass range mass spectrometry, although this comes at the disadvantage of reduced sensitivity. Future combinations of multiplexed imaging‐based methods with mass spectrometry detection will create new capabilities for multimodal analysis. For example, targeted detection of transcripts using RNA‐FISH on the same tissue section after imaging mass spectrometry analyses helped resolve host–microbe interactions (Geier *et al*, 2020). Such applications open an avenue to combine label‐free spatial metabolomics or proteomics with other spatial omics approaches with more reports emerging (preprint: Vicari *et al*, 2023). In parallel, spatial multi‐omics for proteins and small molecules or lipids was demonstrated by a MALDI‐IHC approach for targeted detection of proteins by means of photocleavable mass‐tags conjugated to antibody probes, and another round of imaging mass spectrometry for high‐plex detection of peptide tag *in situ* (Lim *et al*, 2023).

A key future area of is subcellular spatial multi‐omics (Park *et al*, 2022), where integration of morphological cell features from microscopy, highly‐multiplexed subcellular detection of gene and protein expression, and metabolite and lipid localizations would be needed. While microscopy‐based approaches for spatial profiling of gene and protein expression are typically the most straightforward for achieving higher resolution without sacrificing sensitivity, further complementation with emerging subcellular technologies for spatial barcoding and subcellular MALDI‐imaging approaches in spatial metabolomics, lipidomics, and soon glycomics can provide interesting new dimensions.

### Artificial intelligence and machine learning

Currently, spatial omics benefits from the rapidly growing computational developments in particular in Artificial Intelligence and Machine Learning (AI/ML), including via bespoke methods, as outlined above. It is easy to imagine a situation in the future in which, in return, spatial omics will enable novel developments in computational biology. Indeed, predicting complex biological systems needs big, diverse, and representative data properly annotated with a desired class, state, or phenotype especially when using machine learning. Here, spatial omics can be a game changer by providing more data than what is possible to collect by bulk omics and with the native context information, and potentially in an easier, cheaper, or faster manner from a large number of cells as compared to the matching single‐cell omics. Taking into account the ongoing revolution of generative machine learning models (Lopez *et al*, 2020; preprint: Cui *et al*, 2023), spatial omics combining location and molecular profiles can serve as a perfect source of large data to train future generative machine learning models predicting cell images, phenotypes, and states.

### Modeling

Besides providing data for future AI/ML, we expect spatial omics to become a major enabler for dynamic mechanistic modeling. Bottom‐up, mechanistic modeling of intracellular processes already benefits from the availability of bulk and, as of recently, single‐cell data (Garrido‐Rodriguez *et al*, 2022; Hrovatin *et al*, 2022). At the same time, top‐down models describing the physiology of organisms at a macroscopic level typically lack molecular characterization with spatial resolution. Here, spatial omics have the potential to provide the missing molecular data to bridge top‐down and bottom‐up modeling approaches, providing valuable information about subcellular compartmentalization, cell–cell interactions, and molecular factors controlling these interactions, molecular tissue architecture, and chemical microenvironment. This will enable building models that span across biological scales, and integrate intracellular processes and physiological responses. These models could then be used to simulate the effect of molecular interventions, such as a mutation or a drug treatment. The large size of this data and the need to account for spatial dimensions in modeling will likely require using high‐performance computing and thus, availability of the next‐generation computing resources for life scientists. These models will probably be first applicable to simpler systems (organoids, organs‐on‐a‐chip, 3D printed organs, and xenografts) that can be used as proxies of complex organs. Expanding spatial omics into 3D to support these complex models will provide new ways to characterize them better and improve their accuracy. It will also offer the possibility to perform extensive manipulation of the system under controlled conditions, potentially transforming drug screening and personalized therapy development. Thereby, spatial omics will be critical to develop a virtual cell representing a real biological cell in its full complexity and heterogeneity and encompassing possible cellular and molecular programs is a long‐demanded yet ambitious future aim, and on the long term, virtual tissues, virtual organs, virtual organisms (including digital twins in medicine), and virtual ecosystems. Spatial omics datasets and atlases that are being generated, in particular from major systematic efforts, can provide information for creating such virtual biological systems as well as for validating their ability to mimic real systems. We further expect that efforts such as Allen Cell Explorer (Viana *et al*, 2023) and Human Protein Atlas (Uhlén *et al* 2015) would be important contributions to populate virtual cells with subcellular organizational details and to explore this information in 3D.

### Clinical and beyond

From a clinical perspective, spatial omics provides molecular data that can revolutionize histology and pathology, which currently exploits conventional stainings (e.g., hematoxylin and eosin; H&E) or individual markers stained with immunohistochemistry followed by microscopy and statistical and machine learning. This is particularly demanding since the conventional staining and microscopy approaches can lack resolution in the disease stages or types. On the contrary, multiple large‐scale characterization studies by spatial omics revealed the depths of spatial and cell heterogeneity in various indications including cancer (Lewis *et al*, 2021) and human myocardial infarction (Kuppe *et al*, 2022). Moreover, spatial omics already helped pinpointing specific tissue architecture, cell composition, and functional states of cells associated with the therapy response (Zhao *et al*, 2019; Helmink *et al*, 2020). Thus, using rich molecular information far exceeding that provided by cytochemistry and histochemistry staining can enable an improved classification and stratification of patients and eventually lead to an improvement of treatments and enable precision medicine. Although it is still early days for such approaches, the first projects following this strategy have already provided promising preliminary results (Irmisch *et al*, 2021). At the same time, machine learning models can predict spatially resolved single‐cell profiles from H&E images, strengthening the link between the phenotype and omics profiles (preprint: Comiter *et al*, 2023). However, it remains to be seen which cellular programs, or intensities of genes, proteins, or metabolites can be predicted and how this information can be used in applications. Furthermore, spatial transcriptomics and histology can be merged using deep learning to combine the molecular coverage of the former with the spatial resolution of the later (Bergenstråhle *et al*, 2022). Overall, spatial omics carry promise for addressing clinical questions already in these early days while the technologies are still maturing (Liu *et al*, 2022; Zhang *et al*, 2022). The search is open for finding the best‐fitting applications and for improving technologies with respect to their scalability, cost, and robustness.

Spatial omics methods are also poised to reveal novel insights into the role and regulation of the microbiome and the human ecosystems by allowing *in situ* omics investigation of hosts and microbes and microbial communities (Tropini *et al*, 2017; preprint: Lötstedt *et al*, 2022; preprint: Saarenpää *et al*, 2022). Similarly, toxicology, infection biology, and microbial pathogenesis fields would benefit greatly from spatial omics approaches, especially at a high resolution (Rendeiro *et al*, 2021; Lempke *et al*, 2023). Although the initial applications were largely focused on tissue samples from mouse models and human donors *en route* to clinical implementations, we expect that spatial omics methods will be increasingly used for environmental samples and nonmodel organisms, and offer new insights for planetary biology and ecosystems (Liang *et al*, 2018; Cao *et al*, 2023).

## Conclusions

With this multifold promise of spatial omics addressing the growing demand for investigating biology in its spatial context, and fueled by the recent technological progress and breakthroughs, the rapid progress of technological developments in this cutting‐edge field is gathering steam. Similar to the ongoing merging of imaging‐focused and sequencing‐focused efforts in spatial transcriptomics, one can imagine spatial omics getting more closely integrated into the broader field of omics. A further convergence will likely be happening between spatial and single‐cell omics with the continuing increase of the spatial resolution across all spatial omics. This can lead to a scenario where the instrumentation and methodological differences between spatial vs. single‐cell vs. bulk omics are blurred or even nonexisting. This would create better opportunities for investigating biology and addressing medical challenges with future “omics” encompassing spatial, single‐cell, and subcellular capacities where even using the term “spatial omics” would sound as strange as “spatial microscopy.”

## Author contributions

**Theodore Alexandrov:** Conceptualization; writing – original draft; writing – review and editing. **Julio Saez‐Rodriguez:** Conceptualization; writing – original draft; writing – review and editing. **Sinem Saka:** Conceptualization; writing – original draft; writing – review and editing.

## Disclosure and competing interests statement

JS‐R has received funding from GSK, Pfizer and Sanofi and fees/honoraria from Travere Therapeutics, Pfizer, Grunenthal, Stadapharm and Astex. SKS is an inventor on patent applications related to some of the DNA barcoding and imaging methods described here, is a consulting scientific co‐founder and shareholder for Digital Biology, Inc., and receives research funding from Cellzome, a GSK company. TA is an inventor of several patents in the field of imaging mass spectrometry and leads a startup‐in‐incubation in the area of single‐cell metabolomics at the BioInnovation Institute.
